# Automated preparation of biological samples prior to high pressure liquid chromatography: Part I- The use of dialysis for deproteinizing serum for amino-acid analysis

**DOI:** 10.1155/S1463924685000396

**Published:** 1985

**Authors:** D. C. Turnell, J. D. H. Cooper

**Affiliations:** Department of Biochemistry Coventry and Warwickshire Hospital Stoney Stanton Road Coventry CV1 4FH UK


Journal of Automatic Chemistry/*Journal of Clinical Laboratory Automation*, Vol. 7, No. 4 (October–December 1985), pp. 177–180

### Automated preparation of biological samples prior to high pressure liquid chromatography: Part I – The use of dialysis for deproteinizing serum for amino-acid analysis

**D. C. Turnell and J. D. H. Cooper**

*Department of Biochemistry, Coventry and Warwickshire Hospital, Stoney Stanton Road, Coventry CV1 4FH, UK*

### Introduction

All methods for the analysis of amino-acids in serum by column liquid chromatography require initial deproteinization of the sample. This is typically performed using sulphosalicylic or perchloric acids for ion-exchange chromatography [1], or acetonitrile when using reversed phase chromatography [2]. However, these techniques are difficult to automate without the use of sophisticated robots.

Although deproteinization using dialysis should not, in principle, suffer from these disadvantages, the resulting dialysate would contain sub-analysable concentrations of amino-acids. However, the high sensitivity of pre-column o-phthalaldehyde/2-mercaptoethanol (OPA/MCE) derivatization and reversed phase high pressure liquid chromatography (HPLC) methods [2–4] permitted the examination of the potential of this deproteinization technique.

This paper describes the use of dialysis for the deproteinization of serum samples for amino-acid analysis and its incorporation into an automated pre-column derivatization HPLC system [5].

### Materials and methods

### *Instrumentation*

The gradient HPLC used comprised an Altex 420 system, a 150 × 4.6 mm column prepacked with 5 μm Ultrasphere ODS (Beckman-RIIC Ltd, High Wycombe, UK) and a Schoeffel FS970 fluorescence detector (Kratos, Manchester, UK) using an excitation wavelength of 240 nm and an emission cut-off filter of 417 nm. Sample injection was performed with a pneumatically operated Rheodyne 7010 valve (Anachem Ltd, Luton, UK) fitted with a 50 μl loop. Chromatographic data was processed by a SP4100 computing integrator (Spectra-Physics Ltd, St Albans, UK). The gradient program and HPLC method have been previously described [2].

### Dialysis and derivatization system

The derivatization system (figure 1) comprised a modified auto-analyser (AA) sampler II, an AAI pump and a 6 in AAII dialyser block fitted with a C-type cuprophane membrane, which has a molecular weight cut-off of 1000 (Technicon, Basingstoke, UK). The sampler was fitted with a double lumen probe that was constructed as previously described [5]. The autosampler was modified by removing the timing cam and motor accessory and replacing the associated microswitch with a miniature open single pole change-over 5 V relay (Radio Spares, London) connected to one of the six external event outputs (T2-T8) of the SP4100. The power supply to the injection valve actuator and the AA1 pump were also controlled by the SP4100 external event outputs via miniature relays. All the relays were fitted with clamping diodes to avoid transistor overload. A BASIC program in the integrator refers to the run time clock to switch the external event outputs during the process cycle (figure 2), starts the Altex 420 gradient programmer following injection and halts the system after the required number of samples have been analysed.

### Reagents

All amino-acids, iodoacetic acid and 2-mercaptoethanol were obtained from Sigma London Chemical Company

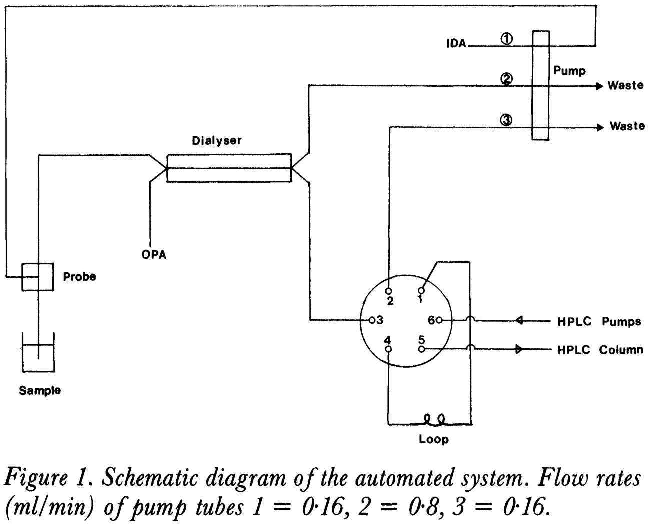

*Figure 1. Schematic diagram of the automated system. Flow rates (ml/min) of pump tubes 1 = 0·16, 2 = 0·8, 3 = 0·16.*

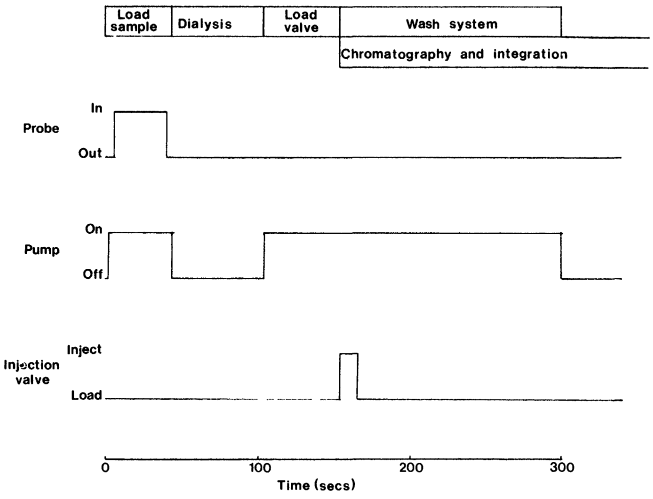

*Figure 2. Timing diagram of the operational sequence of events in the automated system.*

Ltd, Poole, UK. Acetonitrile Far UV grade was obtained from Fisons Scientific Apparatus, Loughborough, UK. Unless otherwise stated, all other chemicals were analytical grade and were supplied by BDH Chemicals Ltd, Poole, UK. Water purified through activated carbon and an ion-exchange resin (Spectrum C system, Elga Ltd, High Wycombe, UK) was used for all reagent preparations.

### *OPA/MCE*

100 mg of OPA was dissolved in 10 ml of methanol and diluted 100 ml with 200 mmol/l sodium borate buffer solution (pH 9.5). To this was added 200 μl of MCE and the reagent stored in a brown glass bottle.

### *Iodoacetic acid*

5.0 g of iodoacetic acid was dissolved in approximately 80 ml of sodium borate buffer solution (200 mmol/l, pH 9.5) and the pH adjusted to 9.5 with 4 mol/l sodium hydroxide solution.

### *Standard amino-acid solution*

The amino-acid standard was an aqueous 500 μmol/l solution of each amino-acid listed in figure 3.

### *Internal standard solution*

The stock internal standard was an aqueous 10 mmol/l solution of homocysteic acid, homoserine and norvaline. Aliquots of both the standard and internal standard solutions were stored at −20 °C. Working internal standard solution was prepared by diluting 1 ml of the stock solution to 50 ml with water; 200 μl of MCE was added to this.

### Procedures

### *Sample preparation*

For routine procedures, 50 μl of serum was added to 500 μl of working internal standard solution and placed in a

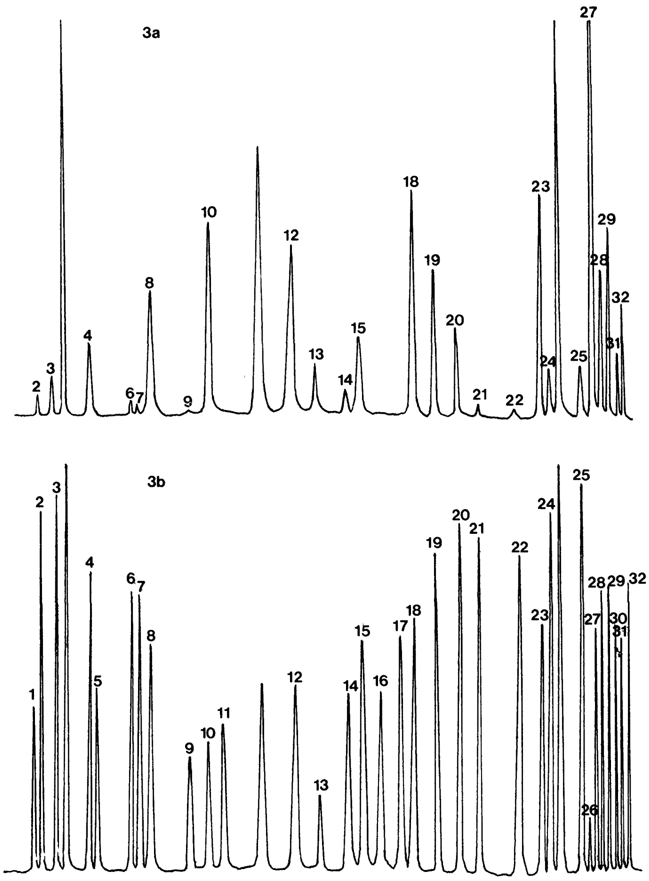

*Figure 3. Chromatograms of (a) a serum from a one-month-old child suffering from hyperphenylalanaemia. (b) An aqueous standard containing 500 μmol/l of each amino-acid. Both chromatographs were obtained using the automated dialysis procedure. The amino-acids are identified as follows: 1, phosphoserine; 2, aspartic acid; 3, glutamic acid; 4, cystine derivative; 5, α-aminoadipic acid; 6, asparagine; 7, homocystine derivative; 8, serine; 9, histidine; 10, glutamine; 11, ethanolamine phosphoric acid; 12, glycine; 13, threonine; 14, citrulline; 15, arginine; 16, 3-methylhistidine; 17, β-alanine; 18, alanine; 19, taurine; 20, tyrosine; 21, α-amino-n-butyric acid; 22, ethanolamine; 23, valine; 24, methionine; 25 tryptophan; 26, L-cystathionine; 27, phenylalanine; 28,L-isoleucine; 29, leucine; 30, hydroxylysine; 31, ornithine; 32, lysine. IS1 = homocysteic acid, IS2 = homoserine, IS3 = norvaline.*

sample cup on the auto-sampler. MCE in the internal standard solution reduces cystine and homocystine to cysteine and homocysteine respectively.

### *Derivatization system*

The configuration of the dialysis and derivatization system is shown in figure 1. The timing diagram for a typical cycle for treating one sample is shown in figure 2 and operates as follows: the integrator initiates the sampler to introduce the double lumen probe into the sample. Sample is aspirated together with iodoacetic acid, which acetylates sulphydryl containing amino-acids [6]. After aspiration of the sample the pump is switched off, the probe is returned to the wash on the sampler and the amino-acids are allowed to diffuse across the dialyser membrane into the OPA/MCE reagent. One minute later the OPA/MCE pump is energized and the amino-acid OPA/MCE derivatives are moved into the injection loop. The injection loop is then switched from load to inject and the derivatized amino-acids eluted onto the column. After 10 s the injection valve is switched to load, and the residual derivatives and sample are purged from the system. The system then shuts down to await the end of the chromatography and integration report.

### Chromatographic conditions and quantitation

The chromatographic conditions used have been described previously [2]. Amino-acid derivatives were identified by their relative retention times and quantified by comparing their peak areas with that for homocysteic acid (internal standard).

### System design and validation

*Process times*

The times of events in the process cycle depend on the following: (1) dialyser size or volume; (2) volumes of connecting tubing; (3) pump tube flow rates; (4) size of injection loop; and (5) analytical recovery of analytes.

The volume of the donor and recipient channels of the 6 in dialyser block was 120 μl and 0.8 mm I.D. teflon tubing was used for all connections. With a 50 μl injection valve loop, and pump tubes of the size indicated in figure 3, the travel of a sample slug through the system was assessed by introducing a small air segment into the sample and recipient sides of the system with the pumps energized and timing their progress. The time for an air segment to travel from the tip of the sample probe to the inlet (donor side) of the dialyser was 6 s, from the inlet to the outlet of the dialyser it was 13 s, and from the outlet of the dialyser (recipient side) to the outlet of the injection loop the time was 30 s. From these figures, it is possible to determine the time required to completely fill the dialyser and the moment at which the Rheodyne valve should be switched from load to inject. The effect of varying the dialysis time on the areas of the chromatographed derivatives of homocysteic acid and serine is shown in figure 4. Serine was used due to the high stability of its OPA/MCE derivative [7].

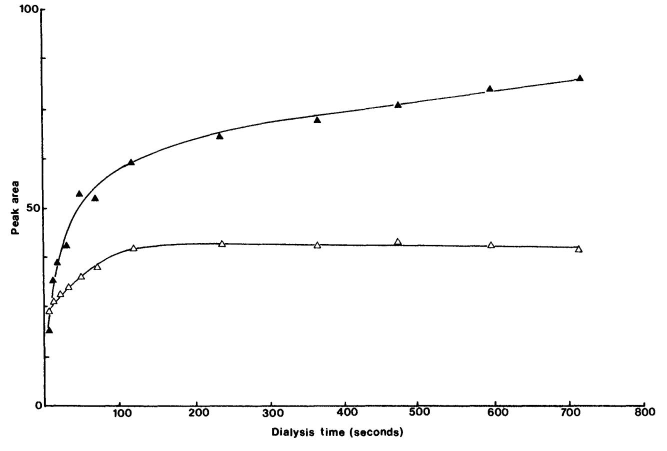

*Figure 4. The effect of dialysis time on the recovery of homocysteic acid ▲——▲ and serine △——△.*

*Precision*

The within- and between-run assay variances of the analysis of serine, threonine, tyrosine and methionine in both an aqueous standard and pooled serum are shown in table 1.

*Accuracy*

The mean analytical recovery was 101% (SD 2·3%), as estimated from analysis of samples supplemented with known quantities of amino-acids. Analytical linearity was only lost if the fluorimeter response exceeded 1·5 times full-scale deflection. However, since quantitation is based on peak area and not peak height, the linearity maxima for each amino-acid depends on both the peak shape and the specific fluorescence intensity of the OPA/MCE derivative. The method was linear with concentration up to a minimum of 1800 μmol/l.

*Matrix interference*

The magnitude of matrix interference was determined by investigating the recovery of homocysteic acid (not usually present in normal serum) from specimens with various concentrations of serum. Serial dilutions of a pooled serum with water were supplemented with the same volume of homocysteic acid solution (1.0 mmol/l). An aliquot of the same pooled serum was supplemented with the homocysteic acid solution and then serially diluted with water. These samples were loaded directly onto the autosampler omitting pre-dilution with internal standard solution and the areas of the homocysteic acid peaks estimated. The peak areas obtained from analyzing these samples are shown in figure 5. The linear responses obtained indicate that the concentration of serum does not affect the recovery of homocysteic acid. With the exception of tryptophan, all the amino-acids listed in figure 3, showed identical response relationships. With serum dilutions of less than one in five, tryptophan exhibited a reduced recovery.

*Dialysis efficiency*

The peak area resulting from known amounts of amino-acids on column was determined by manually derivatizing an aqueous standard solution and injecting onto the HPLC using a filled loop technique (20 μl) with a Rheodyne 7125 valve. The same standard solution was

*Table 1. Precision of assaying a standard solution (500 μmol/l) and a normal serum.*

| | | Within-run $N = 20$ CV% | Between run $N = 20$ CV% |
|---|---|---|---|
| Aqueous standard | Serine | 1·8 | 2·6 |
| | Threonine | 1·9 | 2·9 |
| | Tyrosine | 1·5 | 3·5 |
| | Methionine | 1·1 | 2·4 |
| Serum | Serine | 1·3 | 1·9 |
| | Threonine | 2·5 | 3·5 |
| | Tyrosine | 2·2 | 3·6 |
| | Methionine | 3·8 | 5·5 |

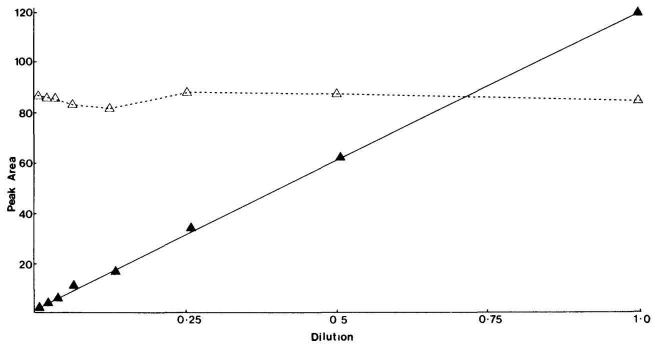

*Figure 5. Peak areas obtained from (a) a diluted serum supplemented with the same amount of homocysteic acid △– – – △; (b) ▲——▲ dilutions of a serum that was initially supplemented with homocysteic acid.*

then dialysed and derivatized using the automated system and the absolute peak areas determined. From these areas it was calculated that 13% of the amino-acids in the sample had passed across the dialyser membrane and derivatized.

### Discussion

Although the pre-column derivatization of amino-acids has been successfully automated [5], the samples still required initial manual deproteinization. The combination of dialysis with the derivatization system provides one way to totally automate sample preparation for amino-acid analysis.

The selected size of the dialyser is dependent on the sensistivity required. Although the use of a large dialyser does not increase the recovery of amino-acids from the sample, if one is used in conjunction with a large injection loop, the amount of amino-acid derivatives loaded onto the HPLC is increased. For precise injection of derivatives, it is essential to use an injection loop with a smaller volume than that of the dialyser channel.

The 60 s dialysis time used does not allow maximum recovery of analytes but yields an adequate sensitivity in a relatively short time. Although at 60 s the recovery of amino-acids is still increasing (see figure 4), the system is quantitatively precise (table 1) due to reproducible process timing via the SP4100 integrator. The effect of serum concentration on the recovery of tryptophan is consistent with protein binding of this amino-acid which has been previously reported [8].

Where relatively large volumes of sample are available, and maximum sensitivity is not required, the process may be simplified by eliminating the stopped flow step, allowing the amino-acids to dialyse and derivatize in a continuous flow mode. Using a smaller OPA/MCE flow rate (0.16 ml/min) than the sample/IDA flow rate (0.8 ml/min) serves to reduce the dilution of derivatives in the dialysate. Counter current dialysis did not improve the observed sensitivity.

The exact process times adopted depend on the flow rates of the pump tubes and volumes of the connecting tubes used. Precise times therefore would need to be established empirically for each individual system.

A major consideration with the design of a sampler for HPLC is the proportion of sample loaded compared with the minimum volume required to be in the sample cup. Ideally, samplers should be able to inject the entire contents of the sample cup, but in practice this is impossible. When the sampler is also required to perform derivatization, a further problem is caused by the small volume of the injection loop relative to the dead volume between the sample cup and the outlet of the injection loop. In this system the load volume: dead volume ratio is maximized by using a double lumen probe and stopped flow dialysis. Using the double lumen probe, sample entering the system is immediately mixed with IDA reagent. The volume of sample aspirated has to completely purge and fill the donor channel of the dialyser, and is dependent on both the size of the dialyser used and the dead volume between the probe and the dialyser inlet. With this system, approximately 37% of the sample aspirated is loaded into the dialyser donor channel, 13% of the amino-acids pass into the recipient channel and 42% of the dialyser channel volume is loaded into the injection valve loop. Thus the equivalent of 2% of the amino-acids sampled is injected onto the HLPC column. However, as the HPLC method has a sensitivity of 38 fmoles on column of each amino-acid [2], samples containing 6 nmol/l of amino-acids may be analysed by the system. The duration of the wash sequence was not optimized with respect to reagent consumption but was adequate to eliminate carry-over. Although excess reagent is consumed, it is insignificant when compared with post-column derivatization where reagent is pumped continually during the chromatography. Furthermore, as the wash sequence occurs while the analytes are being chromatographed, its duration does not influence the time for one total analytical cycle.

By combining automatic dialysis and derivatization, the analysis of amino-acids in complex matrices is facilitated. The system performs sampling, dialysis, derivatization with IDA and OPA/MCE, together with injection onto the HPLC, in only 135 s. Furthermore the efficiency of this clean-up procedure prolongs the life of the analytical HPLC column without employing guard columns.

### References

1. Moore, S., Spackman, D. H. and Stein, W. H., *Analytical Chemistry*, **30** (1958), 1185.
2. Turnell, D. C. and Cooper, J. D. H., *Clinical Chemistry*, **28** (1982), 527.
3. Griffin, M., Price, S. J. and Palmer, T., *Clinica Chimica Acta*, **125** (1982), 89.
4. Hogan, D. L., Kraemer, K. L. and Isenberg, J. I., *Analytical Biochemistry*, **127** (1982), 17.
5. Turnell, D. C. and Cooper, J. D. H., *Journal of Automatic Chemistry*, **5** (1983), 36.
6 Cooper, J. D. H. and Turnell, D. C., *Journal of Chromatography*, **227** (1982), 158.
7. Cooper, J. D. H., Ogden, G., McIntosh, J. and Turnell, D. C., *Analytical Biochemistry*, **142** (1984), 98.
8 McMenamy, R. H., Lund, C. C., Van Marcke, J. and Oncley, J. L., *Arch. Biochem. Biophys.*, **93** (1961), 135.

